# Diagnostic and Therapeutic Benefits of Intra-operative Enteroscopy in Epithelioid Angiosarcoma of the Small Intestine

**DOI:** 10.7759/cureus.34056

**Published:** 2023-01-22

**Authors:** Alfadl A Abdulfattah

**Affiliations:** 1 Internal Medicine, Jazan University, Jazan, SAU

**Keywords:** sarcoma, intestinal resection, intraoperative enteroscopy, gastrointestinal bleeding, intestinal angiosarcoma

## Abstract

Intestinal epithelioid angiosarcoma is an uncommon type of vascular tumor with unusually varied clinical presentations, including non-explained severe gastrointestinal bleeding, anemia, and abdominal pain. A 77-year-old man presented with recurrent severe anemia caused by gastrointestinal bleeding. The diagnosis was jejunal angiosarcoma. The patient had three intestinal resections assisted by explorative intraoperative enteroscopy, which detected multifocal intestinal lesions. Intraoperative enteroscopy is a helpful diagnostic and prognostic approach that detects small intestinal tumors. Intraoperative enteroscopy could improve the outcome of intestinal angiosarcomas.

## Introduction

Angiosarcomas are a group of proliferative and aggressive vascular tumors. They can affect any organ but commonly occur in the skin and subcutaneous tissues [1].

Intestinal epithelioid angiosarcoma is an extremely uncommon type of vascular tumor with an unusual, varied clinical presentation, including non-explained severe gastrointestinal bleeding, anemia, and abdominal pain [2]. It is a very aggressive and locally invasive tumor involving the regional lymph nodes. It mainly affects the proximal jejunum and the ileum. In some patients, it can cause complications such as severe GI bleeding, small bowel obstruction, and intestinal perforations, requiring urgent surgical intervention. [3]. Endoscopic interventions have major diagnostic and prognostic roles in cases of intestinal epithelioid angiosarcomas. The diagnosis is challenging when the tumor is not accessible endoscopically for a biopsy. Both histopathological and immunohistochemical examinations are necessary for accurate diagnosis. Surgery (intestinal resection with wide lymph nodes excision) is the treatment option in most cases of intestinal angiosarcoma [4]. However, the recurrence rate is high, and the prognosis is very poor [5].

We reported a case of a 77-year-old male who presented with several episodes of severe iron deficiency anemia requiring multiple blood transfusions due to two occasions of recurrent intestinal angiosarcoma after two intestinal surgical resections. We now performed IOE-guided objective intestinal resection. The patient underwent complete recovery.

## Case presentation

The patient was a 77-year-old gentleman with a significant cardiac past medical history, including myocardial infarction, atrial fibrillation, and left-side heart failure. He had been hospitalized several times with recurrent acute symptomatic (dyspnea with exertion) microcytic iron deficiency anemia and required several blood transfusions. He denied any history of abdominal pain or gastrointestinal bleeding. He also denied previous radiotherapy, industrial chemicals, smoking, or alcohol exposure. He was allergic to penicillin. Clinical examination including digital rectal exam was unremarkable. Laboratory examination showed microcytic anemia with hemoglobin at 66 g/L and MCV at 66 FL. CT chest, abdomen, and pelvis revealed no abnormalities.

**Figure 1:**
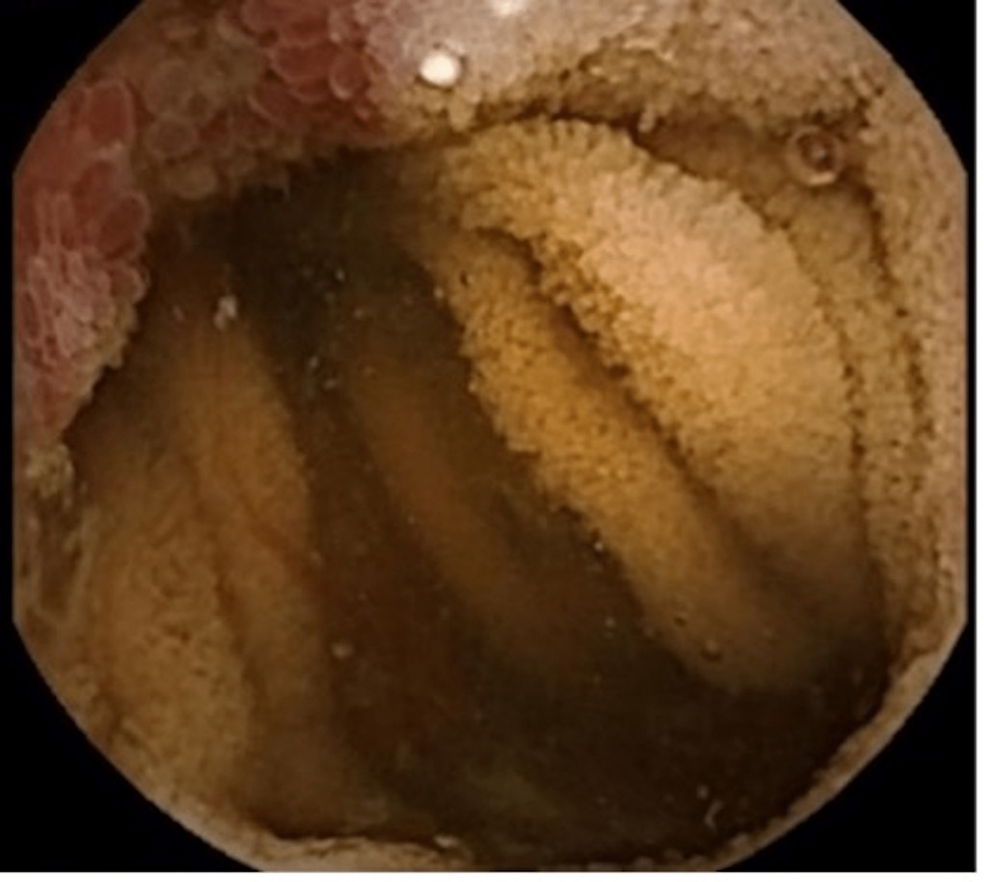
Video capsule shows a nonbleeding ulcerated vascular lesion of the proximal jejunum.

**Figure 2:**
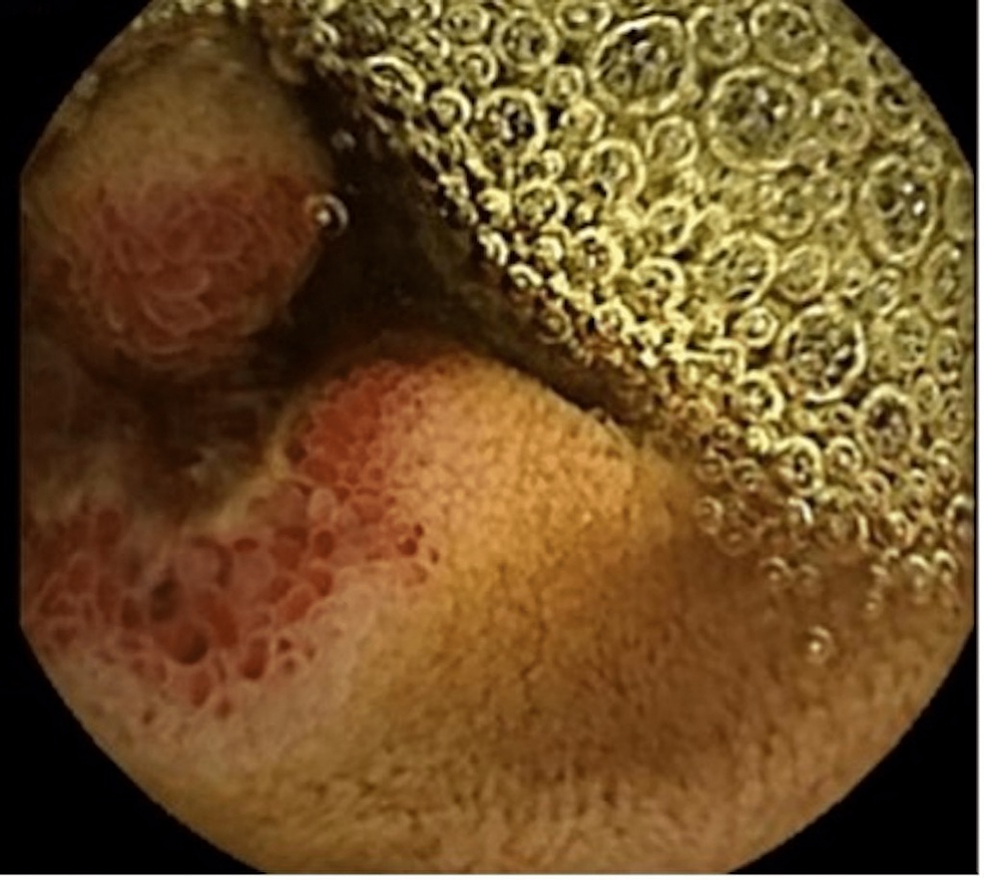
Video capsule shows a necrotic ulcerated jejunal lesion.

**Figure 3:**
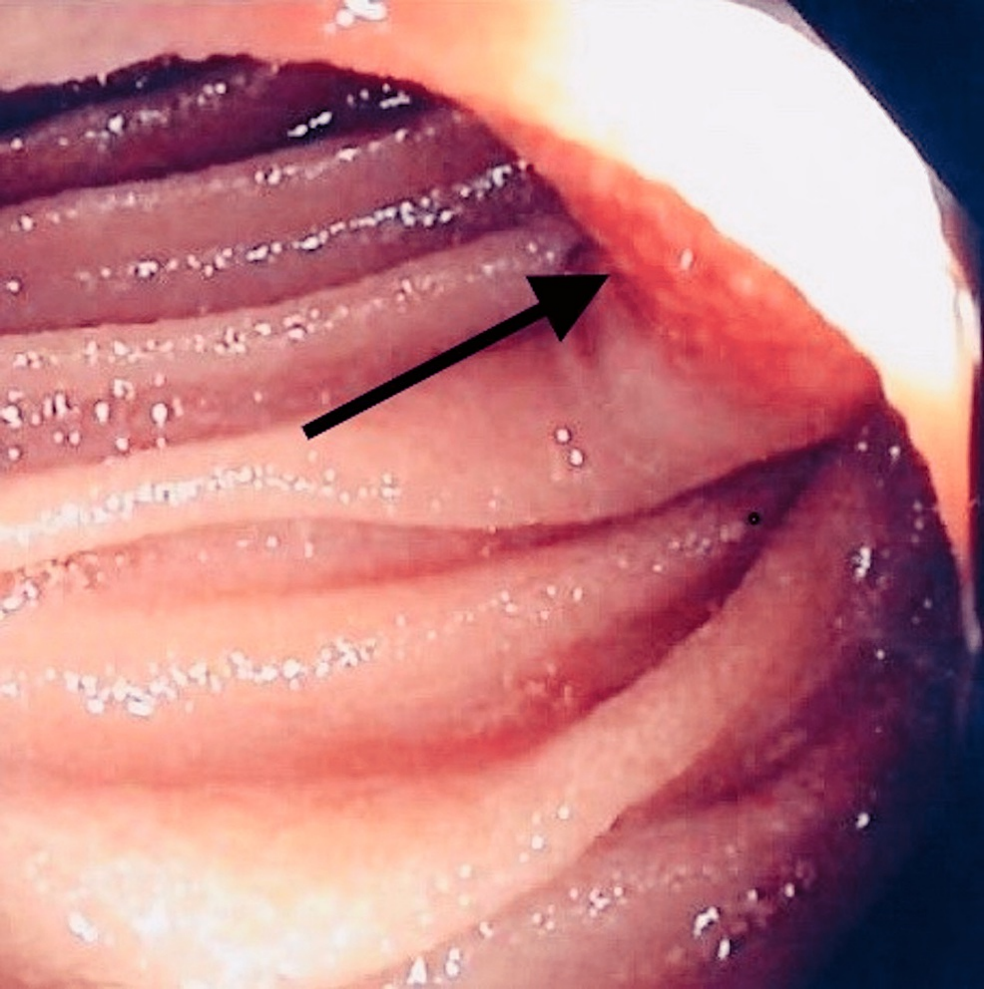
Enteroscopy showed suspected jejunal lesion

**Figure 4:**
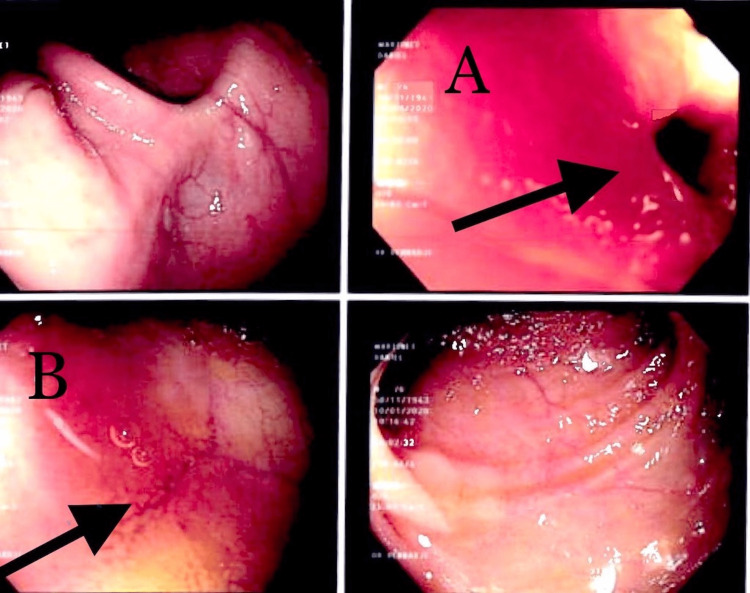
Enteroscopy showed (A) suspected ulcerated jejunal lesion and (B) suspected non-bleeding vascular lesion of proximal jejunum.

Esophagogastroduodenoscopy and colonoscopy did not explain the cause of this presentation. A complementary study with video capsule endoscopy showed the presence of an atypical, suspected nonbleeding ulcerated lesion of the proximal jejunum and other vascular jejunal lesions (Figures 1, 2). Enteroscopy using a duodenoscope, performed under general anesthesia, confirmed an actively bleeding 2-cm jejunal ulcer, posing two marking metallic clips and injection with indigo carmine-ink for eventual resection (Figures 3, 4). The urgent surgical intervention involved the resection of 5 cm of proximal jejunum and end-to-end anastomosis. Histological and immunohistochemistry examinations were satisfactory.

**Figure 5:**
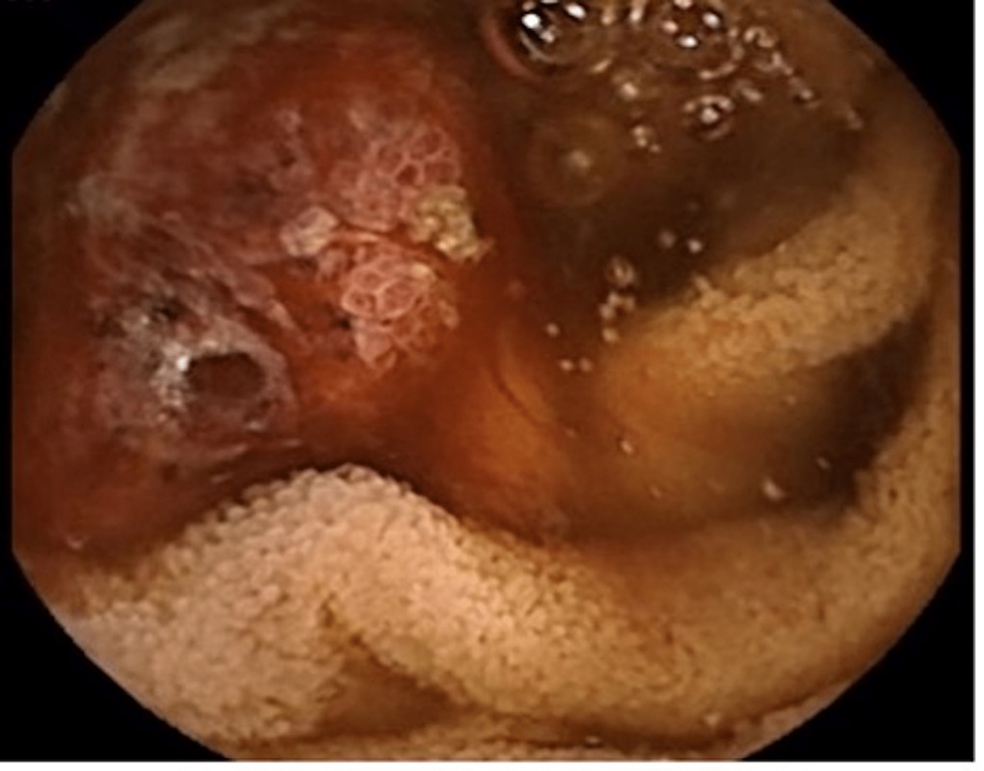
Video capsule showed an active bleeding jejunal lesion.

**Figure 6:**
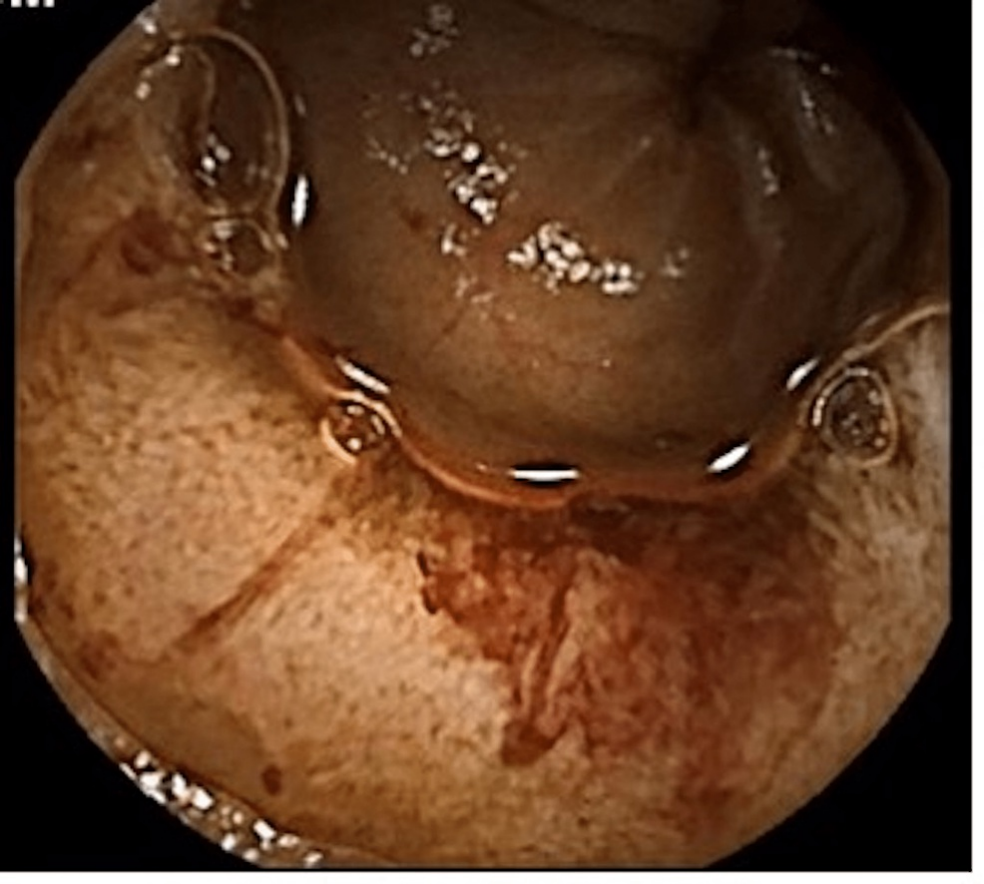
Enteroscopy showed active jejunal bleeding in an ulcerated and anastomosis zone.

Two months later, the patient presented with shortness of breath, melena, and anemia. We performed an urgent video capsule endoscopy that discovered multiple suspected jejunal ulcers actively bleeding (Figures 5, 6).

Emergent surgical laparotomy assisted by gastroenterologist, who performed IOE, revealed two bleeding ulcers 10-15 cm downstream from the site of anastomosis). We resected 20 cm of the small intestine with later-lateral anastomosis and jejunostomy of discharge. Histological and immunohistochemistry examinations revealed epithelioid angiosarcoma of the submucosa of the proximal jejunum, the presence of CD 31 expression, and the absence of CK AE1/3 expression.

After one month, the patient was re-presented with the same symptoms. We performed an urgent laparotomy with intraoperative upstream and downstream enteroscopy from the point of jejunostomy. The upstream enteroscopy detected four scattered ulcers in the proximal jejunum that were biopsied and identified by four metallic clips and indigo carmine injections. Downstream, an enteroscopy was carried into the right colon; no lesions were found. A 30-cm section of the small intestine was resected with extensive lymph-node dissection. Histopathological examination confirmed epithelioid angiosarcoma without lymph node metastases (0/9). The immunohistochemical test was positive for CD 31 and ERG.

Follow-up with laboratory examination one month after the last surgery showed stable hemoglobin. The positron emission tomography scan (upon the tumor board's recommendation) did not show any metabolic activity of the small intestine, and there were no metastatic lesions.

## Discussion

Sarcomas are a group of soft-tissue tumors that vary in prognosis according to subtypes. The most common types are gastrointestinal stromal tumors (GISTs), liposarcoma, leiomyosarcoma, and pleomorphic sarcoma [6]. Angiosarcoma is a rare subtype, accounting for 1%-2% of all soft-tissue sarcomas. It is a vascular lesion and originates from the vascular endothelium. Angiosarcoma can affect any organ; the most common organs are subcutaneous and skin tissues [1]. Most of the reported cases show at least one of the following risk factors; more than ten years of radiotherapy exposure and massive occupational exposure to polyvinyl chloride, vinyl chloride, thorium dioxide, or arsenic [3].

Intestinal epithelioid angiosarcoma is a rare, aggressive, and locally invasive vascular tumor. It mainly affects the proximal jejunum and the ileum. The most common clinical presentation is abdominal pain, obscure or occult gastrointestinal bleeding, anemia, and other nonspecific manifestations such as nausea, vomiting, and weight loss [7]. Acute presentation with gastrointestinal bleeding or melena is not uncommon, requiring treatment by blood transfusions (the typical presentation of our case report) [3]. In some cases, this type of tumor causes serious complications - e.g., small bowel obstruction or intestinal perforation - that necessitate urgent surgical intervention. Intestinal epithelioid angiosarcoma has a very poor prognosis, and the median survival rate is less than one year [8].

Many factors make the diagnosis of intestinal epithelioid angiosarcoma challenging; the similarities of clinical presentations, histopathological similarities with other tumors, and immunohistochemical overlapping with other vascular tumors. However, accurate diagnosis requires histopathological and immunohistochemical examination of the tumor cells. The immunohistochemistry test is positive for CD31, CD34, vimentin, and factor VIII. Molecular studies help detect mutations in the vascular endothelial growth factor receptor-2 (VEGFR2, also known as KDR or Flk-1) [9].

Endoscopic interventions have significant diagnostic and prognostic roles in intestinal epithelioid angiosarcomas. Wireless video capsule endoscopy detects and localizes the lesions and gastrointestinal bleeding [10]. The enteroscopy specialist takes biopsies, localizes the lesions, marks the lesions with metallic clips, and injects the lesions with indigo carmine stains in preparation for eventual surgery. Endoscopic ultrasound evaluates the local invasions of the tumors and lymph node involvements [11].

Intraoperative enteroscopy IOE is a safe and promising intervention that allows endoscopists to explore the small intestine using the endoscope during surgery. This integrated procedure needs a surgeon and a gastroenterologist [12]. Therapeutic applications of this procedure include small intestinal bleeding, small intestine lesions, and tailoring the extent of surgery in CD patients. IOE is necessary to evaluate the tumor's intraluminal extent, detect multifocal intestinal lesions missed by conventional diagnostic tools, and take biopsies of the small bowel lesions [13].

The treatment modalities include Intestinal resection with or without chemotherapy, depending on the cancer stage. However, the risk of cancer recurrence is very high [14]. The risk of recurrence depends on several factors, one of which is the presence of multifocal small intestinal lesions that could not be visualized by conventional endoscopic methods [12]. It has a very poor prognosis, and the median survival rate is less than one year [15].

The IOE could help detect subtle lesions that decrease the risk of recurrence and improve the outcome. In our case, the patient had two occasions of cancer recurrence caused by multifocal intestinal lesions that were not visualized by conventional endoscopy. IOE helped detect subtle lesions and was used for objective intestinal resection, and a normal PET scan proved one-year cancer-free.

Improving survival in such aggressive tumors supports that IOE is a practical approach that could be implicated in various other SB pathologies that require surgical intervention. In a retrospective study, IOE allows recurrence-free management of persistent OGIB in 76% of cases [16]. The advantage of this approach is that it prevents further complicated surgeries caused by cancer recurrences, complex strictures, and persistent bleeding, eventually improving survival and the outcome. The limitation of the IOE approach is that it is a surgical intervention that could risk anesthesia and surgical morbidities.

Although there are no recommendations for using IOE in patients with SB lesions, our findings suggest utilizing IOE in selected cases, including patients who failed conventional therapy or who are undergoing SB surgery; Bonnet S et al. described similar implications: when a preoperative work-up has diagnosed SB lesions that cannot be definitively managed by conventional treatment or whenever surgery is indicated. Given that it is a high-grade and proliferative tumor, we suggest a very close follow-up every two to three months (clinical, radiological, and endoscopic) [15].

## Conclusions

We concluded that using IOE during surgery is a valuable approach for reducing the recurrence rate of intestinal angiosarcoma and improving survival. This finding supports that IOE is a practical technique for cancerous and non-cancerous small bowel lesions that require surgical intervention. Further diagnostic and therapeutic clinical trials and case series are required to learn more about more therapeutic approach.
